# Dietary and Physical Activity Correlates of Muscle Mass in 60–65-Year-Old Seniors: A Gender-Specific Analysis

**DOI:** 10.3390/nu17111930

**Published:** 2025-06-04

**Authors:** Bartłomiej K. Sołtysik, Paweł Balicki, Klaudia Kowalczyk, Aleksandra Lutostańska, Julia Dmuchowska, Małgorzata Pigłowska, Tomasz Kostka

**Affiliations:** Department of Geriatrics and Internal Medicine, Healthy Ageing Research Centre (HARC), Medical University of Lodz, Pomorska Street 251, 92-213 Lodz, Poland; pawel.balicki@umed.lodz.pl (P.B.); k.kowalczyk@csk.umed.pl (K.K.); a.lutostanska@csk.umed.pl (A.L.); j.dmuchowska@csk.umed.pl (J.D.); malgorzata.piglowska@umed.lodz.pl (M.P.); tomasz.kostka@umed.lodz.pl (T.K.)

**Keywords:** muscle mass, nutrition, protein intake, physical activity, seniors

## Abstract

**Introduction:** Sarcopenia and loss of skeletal muscle mass represent major public health concerns in aging populations. Although both diet and physical activity (PA) are recognized as modifiable determinants of muscle mass, their effects may differ by sex. This study aimed to examine dietary and behavioral correlates of muscle mass amongst community-dwelling adults aged 60–65 in Central Poland. **Methods:** The study included 134 women and 138 men. Body composition was assessed using bioelectrical impedance (Maltron Bioscan 920, Essex, UK). Dietary intake was evaluated using a 24 h recall analyzed with Dieta 5.0 software. PA was measured using the Seven-Day Physical Activity Recall Questionnaire and the Stanford Physical Activity Indices. Statistical analysis included bivariate correlations and general linear modeling performed separately for women and men. **Results:** In women, skeletal muscle mass (as a percent of body mass) showed significant positive associations with protein intake per kilogram of body weight, magnesium, phosphorus, and moderate health-related PA. Concomitantly, there was a negative correlation with lipids such as long-chain polyunsaturated fatty acids (PUFA). In multivariate models, protein intake remained the only predictor. In men, only protein intake per kilogram of body weight demonstrated a significant association with muscle mass; no other dietary or PA factors were retained in the model. **Conclusions:** The findings indicate that dietary and behavioral factors influencing muscle mass vary by sex. While muscle mass in women is linked to multiple nutritional and lifestyle factors, men appear primarily responsive to total protein intake. These sex-specific differences may underscore the importance of tailored strategies in sarcopenia prevention.

## 1. Introduction

The demographic shift towards an aging population imposes significant challenges on healthcare systems and demands refined strategies aimed at preserving health and functional independence amongst older adults. A critical component of healthy aging is the maintenance of adequate skeletal muscle mass, which serves as a key determinant of physical performance, functional capacity, and ultimately, survival. Amongst individuals transitioning into older age, particularly those in their sixth and seventh decades of life, skeletal muscle mass emerges as a vital marker of physiological reserve.

Sex-related differences in body composition—specifically in terms of lean body and muscle mass—become especially pronounced in early older age. This period is often marked by the onset of sarcopenia, a progressive and generalized loss of muscle mass and strength. Evidence suggests that a healthy dietary pattern may exert a protective role against sarcopenia in women, a finding not consistently observed in men [1]. Similarly, physical activity (PA) appears to mitigate muscle loss [2], although sex-specific differences in response remain inadequately understood.

Both nutritional intake [3,4,5] and PA [6,7,8] are established as key modifiable determinants of muscle mass. A high-protein diet, traditionally emphasizing animal-derived proteins, is associated with enhanced anabolic responses [9,10,11]. Nonetheless, recent studies indicate that plant-based proteins, when appropriately supplemented—particularly with leucine—may offer comparable anabolic potential [9]. Interestingly, some evidence suggests that plant-based dietary patterns may confer broader functional benefits, such as improved independence [12].

Beyond protein, other dietary components play a crucial role in muscle health. Lipid intake, specifically mono- and polyunsaturated fatty acids [13], as well as dietary fiber [14], has been implicated in modulating sarcopenia risk. However, dietary regimens that exclude high-quality protein sources—such as strict vegan diets—may lack sufficient anabolic properties to support muscle maintenance in older adults [15]. Longitudinal data indicate that a diet rich in vegetables, whole grains, and animal protein is positively associated with muscle mass over time, although such findings are derived from male-only cohorts [16].

Micronutrients also contribute to muscle integrity. According to existing reviews, selenium, calcium, magnesium, and phosphorus are inversely associated with sarcopenia prevalence [17]. Some studies amongst community-dwelling older adults reinforce these associations [18]. While magnesium supplementation shows limited impact on muscle strength in the general population, subgroup analyses indicate potential benefits in magnesium-deficient individuals, particularly seniors [19]. Emerging evidence further supports magnesium’s role in attenuating age-related muscle loss, especially amongst women [20]. Another study indicated a potential effect of magnesium in the preservation of age-related muscle mass loss in a group of women [21]. The notable differences between women and men identified in this study reflect a pattern observed across multiple studies. In one research study conducted in a group of older women, low protein intake had an impact on lower muscle mass but also a higher occurrence of physical limitations [22]. A large Korean cohort study demonstrated an inverse association between healthy diet and low muscle mass in older adults; notably, this relationship was evident only in women, with no significant association observed in men [23].

PA remains the cornerstone of sarcopenia prevention and treatment. Resistance training is particularly effective in mitigating muscle atrophy associated with aging [2,7,24]. In addition, regular engagement in moderate-to-vigorous leisure-time PA appears to confer protective effects [25]. Hormonal changes, especially the decline in estrogen during menopause, significantly contribute to muscle mass loss in women [26], yet similar processes are observed in aging men [27]. Overall, PA is consistently recognized as the most robust non-pharmacological intervention to counteract sarcopenia [8].

Despite growing research in this domain, important gaps remain in understanding the interaction between dietary components, PA patterns, and sex-specific influences on muscle mass dynamics in aging populations.

To our knowledge, this study represents the first attempt to identify factors associated with skeletal muscle mass in a cohort of community-dwelling older adults residing in Central Poland. This area is characterized by particularly unfavorable aging-related health indicators, the highest advancement of the aging process and the highest values of negative health measures in the whole country [28]. Given the escalating public health implications of sarcopenia, especially in regions with limited access to preventive care, continued research in this field is of paramount importance.

## 2. Materials and Methods

The study reports findings from the project titled “The Occurrence of Oxidative Stress and Selected Cardiovascular Risk Factors in Relation to the Functional Status of Older Adults in the Context of Workload” (conducted by the Central Institute for Labor Protection–National Research Institute, Warsaw, Poland). The research involved community dwellers (150 men and 150 women), all age-matched during recruitment, which was conducted via local community centers and senior organizations. A total of 16 women and 12 men were not qualified for further examination because of contraindications to performing bioimpedance analysis (like implantation of a pacemaker or metal prostheses). Participants were independent, community-dwelling volunteers who met the inclusion criteria of being between 60 and 65 years old and providing informed written consent. The study received approval from the Committee on the Ethics of Research in Human Experimentation at the Medical University of Lodz (RNN/648/14/KB, dated 23 September 2014) and was conducted in accordance with the ethical principles outlined in the Helsinki Declaration.

### 2.1. Diet

After analyzing the participants’ diets, the daily intake of individual nutrients was estimated based on a detailed analysis of their menus. Food and beverage consumption on the most representative day was assessed by a qualified dietitian using a 24 h dietary recall questionnaire, supported by a portion size folder containing graphical illustrations of typical portion sizes (e.g., several differently sized servings of the same dish). To minimize reporting errors, participants were asked in advance to prepare a list of all food products, snacks, and beverages consumed during the days of assessment. Dietary intake data were collected over three consecutive days, and the day deemed most representative of the participant’s typical diet was selected for analysis. Importantly, interviewers were instructed not to influence or assess the diet themselves. The reported dietary data were analyzed using Dieta 5.0 software (National Food and Nutrition Institute, Warsaw, Poland), which was used to calculate energy and nutrient intake. This method has also been applied in previous studies [29].

### 2.2. Body Composition

Muscle mass, presented as a percentage of total body mass, was determined using electrical bioimpedance. (Maltron Bioscan 920, Maltron International Ltd., Rayleigh, Essex, UK). Two injector electrodes were positioned on the dorsal side of the foot and wrist, while two detector electrodes were placed between the styloid processes of the radius and ulna, as well as between the medial and lateral malleolus. Throughout the measurement, each participant lay in a supine position with their feet separated and hands resting at their sides [30]. The Maltron BioScan 920-II has demonstrated high reliability in clinical studies, particularly for assessing body composition in older adults [31,32], and in our study, the device was calibrated prior to testing the subjects to ensure measurement accuracy.

### 2.3. PA

The Seven-Day Physical Activity Recall Questionnaire measures weekly sleep hours and categorizes activities into light, moderate, hard, and very hard based on energy expenditure. Energy expenditure was estimated based on activity intensity, assigning specific calorie burn rates per kilogram of body mass per hour for sleep and varying physical activity levels. Each activity category was assigned a specific energy cost expressed in kilocalories (kcal) burned per kilogram of body mass per hour: 1 kcal/kg/hour for sleep, 1.5 kcal/kg/hour for low-intensity activity, 4 kcal/kg/hour for moderate-intensity activity, 6 kcal/kg/hour for vigorous-intensity activity, and 10 kcal/kg/hour for very vigorous-intensity activity. Total weekly energy expenditure was then calculated by multiplying the hours spent in each activity category by its corresponding rate and summing the results [33].

The Stanford Moderate Index assesses health-related PA behaviors through daily habits like taking stairs, walking instead of driving, or parking farther. The Stanford Hard Index evaluates vigorous activities performed regularly for at least three months, such as jogging, cycling, or swimming. These indices, scored from 0 to 6 (moderate) and 0 to 5 (hard), are summarized as PA-health-related behaviors I (PA-HRB I) and II (PA-HRB II), respectively. Standardized protocols are detailed in a previous publication [34].

### 2.4. Statistical Analysis

The statistical analysis of the variables was conducted using both parametric and non-parametric tests. The Shapiro–Wilk test was employed to evaluate the empirical distribution of the variables, while the Levene test was used to assess variance homogeneity. Measures of central tendency, including the arithmetic mean (m) and median (Me), were calculated, along with measures of dispersion such as standard deviation (SD) and the lower and upper quartiles. For quantitative variables, the non-parametric Mann–Whitney U test was used. Additionally, Spearman’s rank correlation test (rho coefficient) was utilized to determine relationships between two quantitative variables. To compare qualitative variables, the chi-square test was used. For variables that showed statistical significance in bidirectional tests, general linear models were built, and non-significant variables were subsequently removed through stepwise elimination. Due to known sex-based differences in body composition and nutritional profiles, separate general linear models were developed for women and men to ensure appropriate interpretation of predictors. All analyses were conducted using Statistica software, version 13.3 (Statsoft, Kraków, Poland). To ensure adequate statistical power for detecting a meaningful association, a priori power analysis was conducted for the correlation test. Assuming a two-tailed significance level of α = 0.05, a desired power of 0.80 (1 − β), and an expected small-to-moderate correlation effect size of r = 0.20, the estimated required sample size was calculated. The analysis indicated that a minimum of 198 participants would be necessary to reliably detect a correlation of this magnitude. The final sample size of 272 participants exceeded this requirement, ensuring sufficient statistical power for the analyses conducted.

## 3. Results

Table 1 presents basic nutritional data and PA parameters stratified by sex, including statistically significant differences between groups. However, the comparison between sexes was not accounted for in the subsequent analysis due to the inherently distinct dietary profiles of women and men, as well as sex-specific patterns of PA influenced by anthropometric differences. All calculated data used for the initial assessment are presented in Supplementary Materials Tables S1 and S2.

Given the number of parameters included in the table, variables that were not utilized in further analyses—due to lack of correlation or absence of effect in the multivariate model—were excluded (presented in Tables S1 and S2). The omitted variables include: animal protein [g], isoleucine [mg], leucine [mg], lysine [mg], methionine [mg], phenylalanine [mg], cystine [mg], tyrosine [mg], threonine [mg], tryptophan [mg], valine [mg], arginine [mg], alanine [mg], aspartic acid [mg], glutamic acid [mg], glycine [mg], proline [mg], serine [mg], histidine [mg], myristoleic acid [c14:1. g], pentadecanoic acid [c15:1. g], palmitoleic acid [c16:1. g], heptadecenoic acid [c17:1. g], oleic acid [c18:1. g], eicosenoic acid [c20:1. g], linoleic acid [c18:2. g], alpha-linolenic acid (ala) [c18:3. g], arachidonic acid (aa) [c20:4. g], eicosatrienoic acid [c20:3. g], butyric acid [c4:0. g], caproic acid [c6:0. g], caprylic acid [c8:0. g], capric acid [c10:0. g], lauric acid [c12:0. g], myristic acid [c14:0. g], pentadecanoic acid [c15:0. g], palmitic acid [c16:0. g], heptadecanoic acid [c17:0. g], stearic acid [c18:0. g], arachidic acid [c20:0. g], sodium [mg], potassium [mg], calcium [mg], iodine [mg], iron [mg], vitamin A [µg], retinol [µg], beta-carotene [µg], vitamin E [mg], riboflavin [mg], niacin [mg], vitamin B6 [mg], vitamin C [mg], vitamin B12 [µg], folate [µg], sucrose [g], lactose [g], cholesterol [mg], energy [kcal], water [g], ash [g] and starch [g].

Table 2 presents the population characteristics based on occupation, residence, and marital status. Women were more frequently divorced, widowed, or single compared to men. Regarding medical history, men more often reported hypertension and myocardial infarction, while women more frequently experienced osteoporosis, gastrointestinal disorders, and depression.

Women were also more likely to use angiotensin II receptor blockers. Among women with hypertension or diabetes mellitus, significantly lower muscle mass was observed (z = −5.05, *p* = 0.001; z = −3.56, *p* = 0.001, respectively). Additionally, women taking anticoagulants, beta-blockers, calcium channel blockers, or angiotensin-converting enzyme (ACE) inhibitors also had significantly lower muscle mass (z = −3.17, *p* = 0.001; z = −3.01, *p* = 0.002; z = −2.75, *p* = 0.005; z = −3.73, *p* = 0.001, respectively).

However, none of these variables remained significant in the general linear model.

Table 3 presents the key correlation matrices between skeletal muscle mass (expressed as a percentage of total body mass) and dietary components, as well as PA variables. In women, skeletal muscle mass was positively correlated with several nutritional and behavioral factors, including protein intake per kilogram of body weight, plant-derived protein, total carbohydrate intake, dietary fiber and digestible carbohydrates. Positive associations were also observed for selected micronutrients such as phosphorus, magnesium, zinc, copper, manganese, as well as for thiamine. Additionally, higher levels of moderate and hard health-related PA were associated with greater muscle mass. Conversely, negative correlations were identified between muscle mass and intake of vitamin D, erucic acid, stearidonic acid, eicosapentaenoic acid (EPA), docosapentaenoic acid (DPA), docosahexaenoic acid (DHA), and long-chain PUFA. In men, skeletal muscle mass showed a positive correlation with protein intake per kilogram of body weight and engagement in hard health-related PA.

In the next step, the variables significant in bivariate analyses were employed in the general linear models (calculated separately for women and men). Although numerous variables correlated with muscle mass, in the general linear model for women, the muscle mass was associated only with intake of protein per 1 kg of body mass [Equation (1)]. For men, the model indicates an association with protein intake per 1 kg of body mass [Equation (2)]. All variables significant in univariate analysis were initially included in the multivariate model, followed by stepwise removal of non-significant ones. Protein intake remained the only consistent dietary predictor, as other nutrients lost significance after adjustment, likely due to collinearity between dietary components.

Equation (1) for muscle mass in women:

Females muscle mass _[% of body mass]_ = 24.11 + 2.49 × Protein per 1 kg of body weight [g/kg], (1)

for protein per 1 kg of body weight [g/kg] *p* < 0.000001; F = 26.048,
for whole model *p* < 0.000001; F = 2240.04,
R^2^ = 0.16.

Equation (2) for muscle mass in men:

Males muscle mass _[% of body mass]_ = 34.19 + 1.90 × Protein per 1 kg of body weight [g/kg], (2)

for protein per 1 kg of body weight [g/kg] *p* = 0.000023; F = 19.276,
for whole model *p* < 0.000001; F = 5049.60,
R^2^ = 0.12.

## 4. Discussion

To our knowledge, this is the first attempt to assess the percentage of muscle mass in a population of young older adults residing in Central Poland, with particular emphasis on the role of diet and PA patterns as contributing factors. The collected data reveal a substantial disparity between sexes, with notable differences observed in both dietary composition and PA, each of which may independently or synergistically influence muscle mass.

In women, muscle mass demonstrated significant associations with multiple dietary and lifestyle variables. According to the general linear model, muscle mass correlated positively with protein intake standardized per kilogram of body weight. However, in correlations, muscle mass was associated with numerous dietary components, such as intake of PUFAs, phosphorus, and magnesium. Additionally, moderate engagement in health-related physical activity appeared to play a contributory role.

When considering dietary components, the female participants who exhibited lower muscle mass tended to consume diets rich in shellfish, nuts and seeds, meat (e.g., liver), legumes, and dairy products. Amongst these, protein intake emerges as the most consistently validated dietary determinant of muscle mass, as confirmed by numerous studies [3,5]. However, ongoing research continues to explore the optimal type, amount, and timing of protein ingestion, particularly with an emphasis on early-day consumption, such as during breakfast, which may enhance anabolic response [35]. Despite this, some meta-analyses suggest no significant differences in outcomes when comparing plant-based versus animal-based protein sources in relation to lean mass or muscle strength [36]. In therapeutic contexts, especially concerning sarcopenia, a daily intake of 1.4 g of protein per kilogram of body weight has been linked to muscle mass preservation [37,38]. This threshold may reflect age-related declines in anabolic sensitivity and the uneven amino acid profiles found in various protein-containing foods, which complicate efficient muscle protein synthesis [39,40]. In our research, there was no differentiation for the source of protein in relation to muscle mass.

PUFAs have also been proposed as protective agents against muscle mass decline. Several studies indicate that PUFA consumption may decrease the risk of low muscle mass in the general population [41] and interventions with PUFA intake seem to improve the gait speed [42,43] or slow down the muscle loss [44]. The properties of fatty acids seem to exhibit anti-inflammatory effects, increase activation of the mTORC1 (mechanistic target of rapamycin complex 1) signaling pathway, and reduce intracellular protein degradation. Additionally, the substances promote mitochondrial formation and function, enhance amino acid transport and uptake, and modulate activity at the neuromuscular junction [45]. A meta-analysis on omega-3 supplementation in older adults supports these findings, showing improved muscle strength and functional performance [43]. Our data, however, in bidirectional analyses, indicate a negative association between some polyunsaturated fatty acids and muscle mass in the group of women. This unexpected finding may reflect reverse causality—where women with lower muscle mass might increase PUFA intake in response to health concerns—or population-specific dietary patterns. The age- and disease-related muscle loss prevention caused by PUFA has been proven [46]. A minimum intake of 2 g/day of omega-3 fatty acid is linked with muscle gain and walking speed improvement [43]. Similarly to PUFA, our study has found a negative correlation between vitamin D3 and muscle mass in women. Again, previously conducted observations indicate a positive association between vitamin D3 and muscle tissue [47] and its deficiency is one of the risk factors for developing sarcopenia [48,49]. In our research, both described relations are presented in women. There are several reasons why the results of this study might differ from the previous research. One possibility is that the people in our study are different from those in the earlier studies—for example, the earlier studies may have looked at individuals who were already at high risk for health problems, while our study involved generally healthy people who may respond differently. Another explanation could be reverse causality—people with lower muscle mass might start taking supplements in an effort to improve their health, which could make it seem like supplement use is linked to lower muscle mass, even though it is actually the result, not the cause. Nonetheless, this result demands further investigation.

In the current study, phosphorus intake was independently associated with muscle mass percentage in women. While phosphorus is primarily recognized for its role in bone health, especially in concert with calcium in the pathophysiology of osteoporosis [50], its high intake has also been linked to adverse renal outcomes [51]. Importantly, phosphorus-rich diets often overlap with high-protein dietary patterns due to their shared sources: animal and plant proteins, fish, seafood, dairy and legumes. Bidirectional analysis showed a strong positive correlation between phosphorus and muscle mass. However, in multivariate models, this relationship is not statistically significant. This may be attributed to collinearity with protein intake, which could mask or confound the independent effect of phosphorus. Alternatively, adjusted models may reflect the role of phosphorus, as some studies have found lower phosphorus concentrations amongst individuals with sarcopenia [17] or sarcopenic obesity [52]. Some hypotheses of a phosphorus- and protein-rich acidic diet contributing to bone resorption, muscle loss, and elevated risks of chronic kidney or liver disease also demand consideration [53]. Further investigation is required to elucidate these associations.

In the presented research, magnesium intake was positively associated with muscle mass, though this relationship was exclusive to the female cohort. According to a systematic review, magnesium correlates with muscle mass, strength, and physical performance and may be inversely associated with the prevalence of sarcopenia [54]. While some meta-analyses have failed to demonstrate a benefit of magnesium in the general population, its role appears more significant in older adults and high-risk subgroups [19]. In hypertensive individuals, dietary—but not supplemental—magnesium was linked to increased muscle mass [55]. Consistent results have been reported in studies from the United Kingdom, where magnesium intake was positively associated with skeletal muscle indices in middle-aged and older populations [56]. The absence of this association in the tested male subgroup remains unexplained and needs further investigation.

PA, particularly resistance training, is one of the most robust modifiers of muscle mass across all ages [2,24]. Through activation of anabolic signaling pathways and mitochondrial biogenesis, physical exercise facilitates skeletal muscle hypertrophy [57]. Nevertheless, aging is accompanied by progressive desensitization to anabolic stimuli—both nutritional and mechanical—which contributes to proteolysis and muscle fiber atrophy [58]. In the current study, a nuanced differentiation in the impact of PA was observed. Leisure-time moderate PA, categorized under health-related behaviors, had a significant association with muscle mass in women; in men, leisure-time hard PA was linked with muscle mass. In contrast, energy-expenditure-related PA, encompassing domestic and occupational activities, showed no such relationship. This relation has been previously established in studies involving middle-aged and older adults [59], and again, in our research, the relationship with PA differs by sex.

Amongst male participants, both bivariate and multivariate analyses yielded more modest associations. Only protein intake per kilogram of body mass remained significant in the general linear model. No other dietary or PA variables showed a significant relationship with muscle mass. This suggests that in males, variability in muscle mass is primarily influenced by total protein intake, with other dietary components playing a subordinate role. Furthermore, existing literature indicates that the source of amino acids may be less relevant in predicting muscle mass outcomes amongst men [4,60].

Beyond biological sex differences in muscle composition, sociocultural influences also shape PA patterns and body image expectations, contributing to distinct lifestyle choices between men and women [61,62]. The findings of the present study imply that amongst 60–65-year-old seniors, diet and PA exert differential effects on muscle mass depending on sex, underscoring the need for sex-specific interventions and recommendations.

### Limitations of the Study

This study has several limitations that should be noted. First, its cross-sectional design does not allow for conclusions about cause and effect. The sample is limited to 60–65-year-old adults from central Poland, which may affect the application of the results to other populations. Two measures of PA were based on questionnaires, which can lead to errors in reporting. However, both tools used—Stanford and Seven-Day Physical Activity Recall—are well-known and have been validated. The assessment of diet may also be biased by incorrect reporting of food amounts or portion sizes. An additional limitation is the absence of biochemical markers of protein metabolism and flexibility testing, which could have provided deeper insights into physiological and functional aspects of muscle health. Lastly, the study group was relatively small, which may reduce the strength of the findings, and even though protein intake was a statistically significant predictor of muscle mass, the explained variance was modest, indicating that other unmeasured factors likely contribute to muscle mass variation.

## 5. Conclusions

This study highlights clear differences between women and men in factors related to muscle mass amongst 60–65-year-old adults in Central Poland. In women, muscle mass was associated with protein intake per kilogram of body weight, as well as with selected nutrients such as polyunsaturated fatty acids, phosphorus, and magnesium. Moderate PA, defined as health-related behavior, also showed a significant link in this group. In men, only protein intake showed a consistent association, with no other dietary factors reaching significance. In terms of activity-related aspects, intensive leisure time PA seems to be connotative. These findings suggest a stronger and more complex role of diet and lifestyle in women, while in men, muscle mass appears to depend more on total protein consumption alone. The collinearity between nutrients, particularly protein and micronutrients, may affect the direction and strength of observed relationships. The sex-based differences likely reflect both biological and behavioral mechanisms. Further research is needed to clarify these patterns and guide effective nutritional and activity-based interventions.

## Figures and Tables

**Table 1:** Comparison between women and men in terms of anthropometry, diet, muscle mass and physical activity.

Variable	Women N = 134		Men N = 138		*p*
	Mean ± SD	Median (Lower-Upper Quartile)	Mean ± SD	Median (Lower-Upper Quartile)	
Age [years]	62.39 ± 1.58	62 (61–64)	62.92 ± 1.71	63 (61–64)	0.008
BMI [kg/m^2^]	28.00 ± 4.58	27.51 (24.79–30.79)	28.14 ± 4.47	27.71 (25.08–30.53)	0.81
Muscle mass [% of body mass]	26.51 ± 2.50	26.48 (24.81–28.37)	36.11 ± 2.49	35.88 (34.79–37.85)	<0.001
Total protein [g]	66.65 ± 24.83	65.88 (49.30–81.36)	83.53 ± 32.12	78.73 (61.23–97.62)	<0.001
Protein per 1 kg of body weight [g/kg]	0.96 ± 0.40	0.91 (0.67–1.22)	1.01 ± 0.46	0.93 (0.73–1.21)	0.9
Plant protein [g]	22.39 ± 8.49	21.45 (15.92–27.60)	28.18 ± 12.70	25.60 (20.09–33.36)	<0.001
Total carbohydrates [g]	213.83 ± 83.44	198.71 (151.31–268.65)	275.43 ± 110.34	253.28 (196.00–328.58)	<0.001
Dietary fiber [g]	19.71 ± 7.91	17.98 (14.54–24.65)	22.00 ± 10.05	19.20 (15.04–27.27)	0.25
Long-chain polyunsaturated fatty acids [g]	0.26 ± 0.78	0.03(0.01–0.11)	0.33 ± 0.92	0.04 (0.0–0.11)	0.49
Digestible carbohydrates [g]	194.18 ± 79.57	179.23(135.09–243.36)	253.51 ± 103.70	230.45 (177.15–306.56)	<0.001
Phosphorus [mg]	1122.80 ± 403.70	1113.69 (864.77–1382.86)	1341.27 ± 501.92	1247.15 (976.12–1635.72)	0.001
Magnesium [mg]	294.23 ± 106.12	268.42 (223.32–352.62)	335.79 ± 129.60	311.02 (240.09–383.03)	0.007
Zinc [mg]	9.11 ± 3.25	8.67 (6.96–10.83)	11.42 ± 4.55	10.44 (8.71–13.63)	<0.001
Copper [mg]	1.17 ± 0.44	1.10 (0.83–1.45)	1.29 ± 0.57	1.12 (0.90–1.54)	0.21
Manganese [mg]	5.07 ± 2.08	4.83(3.59–6.19)	5.66 ± 2.78	5.01 (3.74–7.18)	0.26
Thiamine [mg]	1.11 ± 0.54	0.98 (0.72–1.36)	1.45 ± 0.66	1.30 (0.96–1.84)	<0.001
Vitamin D [µg]	2.84 ± 5.08	1.70 (0.85–2.52)	3.52 ± 3.98	2.23 (1.24–3.66)	0.001
Vitamin B12 µg]	4.65 ± 10.80	2.53 (1.62–3.56)	5.41 ± 14.84	2.89 (1.73–4.01)	0.11
Erucic acid [C22:1. g]	0.20 ± 0.47	0.03 (0.00–0.17)	0.21 ± 0.42	0.04 (0.00–0.23)	0.86
Total monounsaturated fatty acids [g]	20.69 ± 14.87	18.24 (12.03–25.31)	29.32 ± 16.04	26.70 (17.07–37.97)	<0.001
Stearidonic acid [C18:4. g]	0.02 ± 0.06	0 (0–0)	0.02 ± 0.08	0 (0–0)	0.96
Eicosapentaenoic acid (EPA) [C20:5. g]	0.08 ± 0.24	0 (0–0.02)	0.10 ± 0.30	0 (0–0.02)	0.52
Docosapentaenoic acid (DPA) [C22:5. g]	0.03 ± 0.09	0 (0–0.01)	0.03 ± 0.07	0 (0–0.02)	0.28
Docosahexaenoic acid [DHA. C22:6. g]	0.15 ± 0.47	0.02 (0.01–0.08)	0.20 ± 0.56	0.03 (0.00–0.09)	0.56
Total polyunsaturated fatty acids [g]	8.93 ± 7.03	7.55 (4.90–10.74)	11.50 ± 8.29	9.80 (5.83–14.12)	0.001
Saturated fatty acids: total [SFA. g]	21.70 ± 17.71	19.86 (12.77–27.02)	31.59 ± 20.88	26.49 (19.02–39.90)	<0.001
Physical activity–health related behaviors moderate (PA-HRBI)	2.86 ± 1.63	3 (2–4)	2.71 ± 1.69	3 (1–4)	0.41
Physical activity–health related behaviors hard (PA-HRBII)	0.26 ± 0.71	0 (0–0)	0.25 ± 0.69	0 (0–0)	0.98
Physical activity–energy expenditure (PA-EE) [kcal/kg/day]	45.74 ± 7.09	44.55 (39.92–50.50)	44.51 ± 8.09	42.04 (37.71–50.25)	0.07

**Table 2:** Comparison between women and men in terms of marital status, occupation, chronic diseases and medications.

Variable		Women N = 134	Men N = 138	*p*
Marital status	Married	71 (52.9%)	113 (81.8%)	0.001
	Divorced	25 (18.6%)	17 (12.32%)	
	Widowed	28 (20.9%)	4 (2.9%)	
	Single	10 (7.5%)	4 (2.9%)	
Place of residence	Urban	126 (94.0)	126 (91.3%)	0.38
	Rural	8 (5.9%)	12 (8.7%)	
Occupation	White collar	45 (33.6%)	45 (32.6%)	0.82
	Physical	41(30.6%)	47 (34.0%)	
	Unemployed	48 (35.8%)	36 (33.4%)	
Diseases	Arterial hypertension	61 (45.5%)	82 (59.4%)	0.02
	Hypercholesterolemia	95 (70.9%)	87 (63.0%)	0.16
	Diabetes mellitus	15 (11.1%)	18 (13.0%)	0.64
	Coronary artery disease	13 (9.7%)	21 (15.22%)	0.16
	Myocardial infarction	2 (1.5%)	11 (7.9%)	0.01
	Heart failure	20 14.9%)	13 (9.42%)	0.16
	Stroke	6 (4.48%)	5 (3.62%)	0.71
	Chronic lung disease	22 (16.4%)	14 (10.1%)	0.12
	Osteoarthritis	68 (51.1%)	61 (44.2%)	0.25
	Osteoporosis	24 (17.9%)	3 (2.2%)	<0.001
	Gastrointestinal disorders	54 (40.3%)	39 (28.3%)	0.03
	Depression	30 (22.4%)	12 (8.7%)	0.01
	Urinary incontinence	31 (23.1%)	23 (16.7%)	0.18
Medications	Anticoagulants	24 (17.9%)	20 (14.5%)	0.74
	Beta-adrenolitics	39 (29.1%)	37 (26.8%)	0.67
	Calcium channel blockers	10 (7.5%)	20 (14.5%)	0.06
	Angiotensin-converting enzyme inhibitors	30 (22.4%)	35 (25.4%)	0.56
	Angiotensin II receptors blockers	16 (11.9%)	7 (5.0%)	0.04
	Diuretics	27 (20.1%)	24 (17.4%)	0.56
	Statins	24 (17.9%	37 (26.85)	0.07

**Table 3:** Correlation between muscle mass expressed as a percentage of body mass with diet components and physical activity.

Variable	Women N = 134		Men N = 138	
	Muscle Mass [%]	*p*	Muscle Mass [%]	*p*
Total protein [g]	0.08	ns	0.04	ns
Protein per 1 kg of body weight [g/kg]	0.41	<0.001	0.35	<0.001
Plant protein [g]	0.25	<0.01	0.08	ns
Total carbohydrates [g]	0.26	<0.01	0.10	ns
Dietary fiber [g]	0.18	<0.05	0.00	ns
Long-chain polyunsaturated fatty acids [g]	−0.24	<0.01	0.00	ns
Digestible carbohydrates [g]	0.25	<0.01	0.10	ns
Phosphorus [mg]	0.21	<0.01	0.01	ns
Magnesium [mg]	0.30	<0.001	0.00	ns
Zinc [mg]	0.22	<0.01	0.02	ns
Copper [mg]	0.20	<0.01	0.01	ns
Manganese [mg]	0.20	<0.05	0.08	ns
Thiamine [mg]	0.17	<0.05	0.02	ns
Vitamin D [µg]	−0.24	<0.01	−0.09	ns
Erucic acid [C22:1. g]	−0.20	<0.05	−0.07	ns
Total monounsaturated fatty acids [MUFA. g]	−0.01	ns	0.04	ns
Stearidonic acid [C18:4. g]	−0.23	<0.01	−0.07	ns
Eicosapentaenoic acid (EPA) [C20:5. g]	−0.21	<0.05	−0.05	ns
Docosapentaenoic acid (DPA) [C22:5. g]	−0.22	<0.05	−0.01	ns
Docosahexaenoic acid [DHA. C22:6. g]	−0.24	<0.05	−0.04	ns
Total polyunsaturated fatty acids [PUFA. g]	−0.03	ns	−0.05	ns
Saturated fatty acids: total [SFA. g]	−0.02	ns	0.07	ns
Physical activity–health related behaviors moderate (PA-HRBI)	0.23	0.01	0.14	ns
Physical activity–health related behaviors hard (PA-HRBII)	0.19	<0.05	0.20	<0.05
Physical activity–energy expenditure (PA-EE) [kcal/kg/day]	−0.01	ns	0.07	ns

ns—not significant.

## Data Availability

The original contributions presented in this study are included in the article and Supplementary Materials. Further inquiries can be directed to the corresponding author.

## Supplementary Materials

The following supporting information can be downloaded at https://www.mdpi.com/article/10.3390/nu17111930/s1, Table S1: Comparison between women and men in terms of anthropometry, diet, muscle mass and physical activity; Table S2: Correlation between muscle mass expressed as a percent of body mass with diet components and physical activity.
