# Supplementary material for: Dietary and Physical Activity Correlates of Muscle Mass in 60–65-Year-Old Seniors: A Gender-Specific Analysis
Source: Nutrients. 2025 Jun 4;17(11):1930. doi: 10.3390/nu17111930 (PMC12157822; doi:10.3390/nu17111930)
Supplement: Supplementary file 1 [file nutrients-17-01930-s001.zip › nutrients-3637059-supplementary.pdf]

nutrients

MDPI

Supplementary Materials:

Table S1. Comparison between women and men in terms of anthropometry, diet, muscle mass and physical activity.

Variable	Women N=134		Men N=138		*p*
	Mean ± SD	Median (Lower-Upper Quartile)	Mean ± SD	Median (Lower-Upper Quartile)	
Age [years]	62.39±1.58	62 (61-64)	62.92±1.71	63 (61-64)	0.008
BMI [kg/m²]	28.00±4.58	27.51 (24.79-30.79)	28.14±4.47	27.71 (25.08-30.53)	0.81
Muscle mass [% of body mass]	26.51 ±2.50	26.48 (24.81-28.37)	36.11±2.49	35.88 (34.79-37.85)	<0.001
Total protein [g]	66.65±24.83	65.88 (49.30-81.36)	83.53±32.12	78.73 (61.23-97.62)	<0.001
Protein per 1kg of body weight [g/kg]	0.96±0.40	0.91 (0.67-1.22)	1.01±0.46	0.93 (0.73-1.21)	0.9
Animal protein [g]	43.14±22.72	41.26 (26.66-54.60)	55.09±27.18	50.89 (38.22-65.78)	<0.001
Plant protein [g]	22.39±8.49	21.45 (15.92-27.60)	28.18±12.70	25.60 (20.09-33.36)	<0.001
Total carbohydrates [g]	213.83±83.44	198.71 (151.31-268.65)	275.43±110.34	253.28 (196.00-328.58)	<0.001
Dietary fiber [g]	19.71±7.91	17.98 (14.54-24.65)	22.00 ±10.05	19.20 (15.04-27.27)	0.25
Long-chain poly- unsaturated fatty acids [g]	0.26±0.78	0.03(0.01-0.11)	0.33±0.92	0.04 (0.0-0.11)	0.49
Digestible carbohydrates [g]	194.18±79.57	179.23(135.09-243.36)	253.51±103.70	230.45 (177.15-306.56)	<0.001
Potassium [mg]	3036.38±1230.82	2833.48 (2192.61-3903.24)	3567.61±1510.29	3414.89 (2465.41-4342.87)	0.002
Calcium [mg]	607.91±310.68	575.21 (358.21-790.63)	653.06±407.22	588.05 (357.97-866.67)	0.65
Phosphorus [mg]	1122.80±403.70	1113.69 (864.77-1382.86)	1341.27±501.92	1247.15 (976.12-1635.72)	0.001
Magnesium [mg]	294.23±106.12	268.42 (223.32-352.62)	335.79±129.60	311.02 (240.09-383.03)	0.007
Iron [mg]	10.49±4.49	9.63 (7.84-12.36)	12.41±6.43	10.73 (8.43-14.34)	<0.001
Zinc [mg]	9.11±3.25	8.67 (6.96-10.83)	11.42±4.55	10.44 (8.71-13.63)	<0.001
Copper [mg]	1.17±0.44	1.10 (0.83-1.45)	1.29±0.57	1.12 (0.90-1.54)	0.21
Manganese[mg]	5.07±2.08	4.83(3.59-6.19)	5.66±2.78	5.01 (3.74-7.18)	0.26
Iodine [mg]	132.3±74.35	124.61 (75.73-168.15)	155.41±74.01	139.76 (96.97-199.80)	0.002
Vitamin A [µg]	1328.9±2889.93	808.18(471.42-1313.65)	1541.71±3923.32	849.26 (579.47-1334.59)	0.25
Retinol [µg]	713.92±2857.57	256.69(159.75-383.13)	948.25±3893.40	339.88 (194.80-577.70)	0.001
Beta-carotene [µg]	3678.45±3118.05	2896.23(1218.47-5048.29)	3497.41±3180.18	2824.71 (1377.81-4280.88)	0.54
Vitamin E [mg]	8.52±5.99	7.33 (4.97-10.02)	9.66±6.18	7.72 (5.35-12.36)	0.08
Thiamine [mg]	1.11±0.54	0.98 (0.72-1.36)	1.45±0.66	1.30 (0.96-1.84)	<0.001
Riboflavin [mg]	1.54±1.01	1.39 (1.12-1.71)	1.80±1.33	1.56 (1.16-2.08)	<0.02
Niacin [mg]	16.24±9.99	13.85 (8.96-20.03)	21.20±11.21	18.74 (14.22-25.37)	<0.001
Vitamin B6 [mg]	1.64±0.77	1.46(1.08-2.01)	2.01±0.87	1.81 (1.41-2.48)	<0.001
Vitamin C [mg]	101.78±82.48	81.91 (51.75-139.56)	116.18±145.40	64.20 (34.46-136.24)	0.10
Vitamin D [µg]	2.84±5.08	1.70 (0.85-2.52)	3.52±3.98	2.23 (1.24-3.66)	0.001
Vitamin B12 µg]	4.65±10.80	2.53 (1.62-3.56)	5.41±14.84	2.89 (1.73-4.01)	0.11

Folate [µg]	264.42±195.48	232.43 (172.7-291.97)	304.79±263.45	255.34 (188.30-337.96)	0.08
Isoleucine [mg]	3115.53±1246.57	3067.9 (2272.9-3873.2)	3963.97±1560.55	3703.90 (2921.84-4692.40)	<0.001
Leucine[mg]	4924.87±1896.17	4870.33 (3651.55-5959.76)	6224.34±2507.61	5875.19 (4514.22-7602.58)	<0.001
Lysine [mg]	4363.49±1932.16	4277.27 (3147.13-5379.13)	5520.49±2367	5166.70 (3849.31-6716.63)	<0.001
Methionine [mg]	1541.34±630.77	1533.83 (1129.60-1904.30)	1955.43±788.16	1811.65 (1397.06-2340.05)	<0.001
Phenylalanine [mg]	2856.65±1043.69	2831.75 (2141.81-3497.86)	3658.63±1416.74	3443.69 (2633.27-4308.70)	<0.001
Cystine [mg]	927.39±315.56	935.47 (664.06-1121.77)	1214.37±470.39	1158.85 (874.19-1433.26)	<0.001
Tyrosine [mg]	2302.22±905.82	2308.02 (1712.17-2841.46)	2911.96±1153.85	2793.50 (2074.86-3394.40)	<0.001
Threonine [mg]	2647.39±1044.33	2596.32(1949.12-3253.48)	3387.06±1329.70	3137.89 (2432.07-4089.24)	<0.001
Tryptophan [mg]	824.13±331.72	826.69 (611.96-1029.62)	1043.11±409.51	972.12 (767.19-1260.11)	<0.001
Valine [mg]	3714.25±1434.94	3724.86 (2783.53-4594.36)	4670.24±1829.69	4374.86 (3392.69-5516.91)	<0.001
Arginine [mg]	3293.00±1355.28	3136.14 (2375.77-4135.15)	4270.40±1780.03	3862.58 (3124.55-5021.71)	<0.001
Histidine [mg]	1852.72±857.28	1776.38 (1275.91-2335.91)	2363.36±996.75	2195.56 (1646.33-2794.19)	<0.001
Myristoleic acid [C14:1. g]	023±0.31	0.19 (0.11-0.29)	0.30±0.26	0.24 (0.14-0.38)	0.01
Pentadecenoic acid [C15:1. g]	0.06±0.04	0.05 (0.02-0.08)	0.07±0.07	0.05 (0.02-0.10)	0.38
Palmitoleic acid [C16:1. g]	1.20±0.96	0.93 (0.68-1.47)	1.85±1.10	1.66 (1.13-2.24)	<0.001
Heptadecenoic acid [C17:1. g]	0.11±0.07	0.10 (0.06-0.14)	0.15±0.13	0.13 (0.06-0.19)	0.02
Oleic acid [C18:1. g]	18.58±13.31	15.69 (10.63-22.90)	26.31±14.50	23.61 (14.93-34.70)	<0.001
Eicosenoic acid [C20:1. g]	0.27±0.45	0.14 (0.06-0.31)	0.35±0.35	0.25 (0.13-0.45)	<0.001
Erucic acid [C22:1. g]	0.20±0.47	0.03 (0.00-0.17)	0.21±0.42	0.04 (0.00-0.23)	0.86
Total monounsaturated fatty acids [g]	20.69±14.87	18.24 (12.03-25.31)	29.32±16.04	26.70 (17.07-37.97)	<0.001
Linoleic acid [C18:2. g]	7.23±6.11	5.84 (3.88-8.30)	9.61±7.66	7.52 (4.68-11.65)	<0.001
Alpha-linolenic acid (ALA) [C18:3. g]	1.32±1.81	0.85 (0.48-1.31)	1.35±1.30	0.93 (0.62-1.77)	0.06
Stearidonic acid [C18:4. g]	0.02±0.06	0 (0-0)	0.02±0.08	0 (0-0)	0.96
Arachidonic acid (AA) [C20:4. g]	0.10±0.13	0.05 (0.01-0.13)	0.17±0.17	0.13 (0.06-0.20)	<0.001
Eicosatrienoic acid [C20:3. g]	0.00±0.001	0 (0-0)	0.001±0.006	0 (0-0)	0.74
Eicosapentaenoic acid (EPA) [C20:5. g]	0.08±0.24	0 (0-0.02)	0.10±0.30	0 (0-0.02)	0.52
Docosapentaenoic acid (DPA) [C22:5. g]	0.03±0.09	0 (0-0.01)	0.03±0.07	0 (0-0.02)	0.28
Docosahexaenoic acid [DHA. C22:6. g]	0.15±0.47	0.02 (0.01-0.08)	0.20±0.56	0.03 (0.00-0.09)	0.56
Total polyunsaturated fatty acids [g]	8.93±7.03	7.55 (4.90-10.74)	11.50±8.29	9.80 (5.83-14.12)	0.001
Butyric acid [C4:0. g]	0.33±0.29	0.29 (0.14-0.44)	0.39±0.40	0.31 (0.17-0.46)	0.35
Caproic acid [C6:0. g]	0.24±0.19	0.21 (0.11-0.32)	0.30±0.28	0.24 (0.14-0.36)	0.09
Caprylic acid [C8:0. g]	0.20±0.29	0.15 (0.09-0.24)	0.24±0.23	0.19 (0.11-0.30)	0.01
Capric acid [C10:0. g]	0.50±0.69	0.37 (0.19-0.64)	0.63±0.71	0.44 (0.28-0.78)	0.02
Lauric acid [C12:0. g]	0.81±1.40	0.56 (0.33-0.90)	0.97±0.86	0.80 (0.43-1.30)	0.003
Myristic acid [C14:0. g]	2.62±2.89	2.18 (1.15-3.34)	3.69±3.24	2.69 (1.73-4.66)	<0.001
Pentadecanoic acid [C15:0. g]	0.31±0.41	0.25 (0.14-0.38)	0.46±0.40	0.34 (0.19-0.61)	<0.001
Palmitic acid [C16:0. g]	11.79±9.13	10.18 (7.45-14.54)	17.35±10.94	14.56 (10.58-22.26)	<0.001
Heptadecanoic acid [C17:0. g]	0.20±0.29	0.16 (0.10-0.24)	0.26±0.21	0.21 (0.12-0.34)	<0.001
Stearic acid [C18:0. g]	4.54±3.50	3.91 (2.50-5.27)	7.05±4.47	6.0 (4.09-9.20)	<0.001
Arachidic acid [C20:0. g]	0.07±0.09	0.05 (0.01-0.08)	0.08±0.07	0.05 (0.02-0.11)	0.08

Saturated fatty acids: total [SFA. g]	21.70±17.71	19.86 (12.77-27.02)	31.59±20.88	26.49 (19.02-39.90)	<0.001
Sucrose [g]	38.55±30.73	29.66±16.82-52.10)	43.71±37.05	32.22 (15.62-58.57)	0.59
Lactose [g]	8.72±7.76	7.00 (2.16-13.32)	9.69±9.87	6.11 (1.39-15.59)	0.97
Sodium [mg]	3017.27±1422.20	2802.29 (2156.76-3842.64)	4155.08±1576.46	3966.02 (3074.55-4882.07)	<0.001
Cholesterol [mg]	253.62±208.12	214.79 (122.87-308.96)	337.31±255.20	263.66 (172.69-431.40)	<0.001
Energy [kcal]	1549.61±579.54	1474.74 (1151.18-1879.42)	2089.27±778.47	1959.44 (1515.95-2558.80)	<0.001
Water [g]	2168.28±775.87	2098.36 (1572.57-2559.00)	2367.50±872.20	2215.12 (1721.34-2891.22)	0.06
Ash [g]	15.01±5.68	14.04 (11.62-18.54)	18.57±6.29	17.83 (13.74-22.11)	<0.001
Alanine [mg]	3135.18±1381.60	3022.97 (2169.55-3902.14)	3973.49±1661.83	3735.49±2772.42-4879.54)	<0.001
Aspartic acid [mg]	5929.68±2332.89	5893.33 (4343.76-734.91)	7509.95±3123.69	6709.33 (5406.36-9176.14)	<0.001
Glutamic acid [mg]	12466.71±4447.69	12584.20 (9235.07-15082.10)	16110.24±6188.14	153430.99 (11981.69-18879.60)	<0.001
Glycine [mg]	2826.41±1488.10	2662.97 (1922.04-3389.19)	3591.17±1761.44	3320.99 (2581.28-4225.30)	<0.001
Proline [mg]	4571.67±1790.61	4585.69 (3430.05-5551.21)	5831.89±2501.32	5486.19(4198.43-6717.04)	<0.001
Serine [mg]	105.24±47.91	97.25 (71.22-133.46)	3935.44±1580.03	3678.28 (2858.09-2661.99)	<0.001
Starch [g]	105.24±47.91	97.25 (71.22-133.46)	154.43±65.30	145.92 (110.73-188.72)	<0.001
Physical activity- Health related behaviours moderate (PA-HRBI)	2.86±1.63	3 (2-4)	2.71±1.69	3 (1-4)	0.41
Physical activity- health related behaviours hard(PA-HRBII)	0.26±0.71	0 (0-0)	0.25±0.69	0 (0-0)	0.98
Physical activity- Energy expenditure PA-EE [kcal/kg/day]	45.74±7.09	44.55 (39.92-50.50)	44.51±8.09	42.04 (37.71-50.25)	0.07

Table S2. Correlation between muscle mass expressed as a percent of body mass with diet components and physical activity.

	Women N=134		Men N=138	
	Muscle mass [%]	p	Muscle mass [%]	p
Total protein [g]	0.08	ns	0.04	ns
Protein per 1kg of body weight [g/kg]	0.41	<0.001	0.35	<0.001
Animal protein [g]	-0.01	ns	0.01	ns
Plant protein [g]	0.25	<0.01	0.08	ns
Total carbohydrates [g]	0.26	<0.01	0.10	ns
Dietary fibre [g]	0.18	<0.05	0.00	ns
Long-chain poly- unsaturated fatty acids [g]	-0.23	<0.01	0.00	ns
Digestible carbohydrates [g]	0.25	<0.01	0.10	ns
Potassium [mg]	0.16	ns	-0.02	ns
Calcium [mg]	0.14	ns	0.10	ns
Phosphorus [mg]	0.21	<0.01	0.01	ns
Magnesium [mg]	0.30	<0.001	0.00	ns
Iron [mg]	0.17	ns	-0.05	ns
Zinc [mg]	0.22	<0.01	0.02	ns
Copper [mg]	0.20	<0.01	0.01	ns
Manganese[mg]	0.20	<0.05	0.00	ns
Iodine [mg]	-0.06	ns	0.06	ns

Vitamin A [µg]	-0.03	ns	-0.12	ns
Retinol [µg]	-0.02	ns	-0.12	ns
Beta-carotene [µg]	-0.03	ns	-0.01	ns
Vitamin E [mg]	0.03	ns	-0.06	ns
Thiamine [mg]	0.17	<0.05	0.00	ns
Riboflavin [mg]	0.06	ns	-0.07	ns
Niacin [mg]	0.09	ns	-0.05	ns
Vitamin B6 [mg]	0.14	ns	-0.05	ns
Vitamin C [mg]	-0.07	ns	-0.13	ns
Vitamin D [µg]	-0.24	<0.01	-0.09	ns
Vitamin B12 µg]	-0.07	ns	-0.13	ns
Folate [µg]	0.04	ns	-0.10	ns
Isoleucine [mg]	0.07	ns	0.02	ns
Leucine[mg]	0.08	ns	0.02	ns
Lysine [mg]	0.04	ns	0.03	ns
Methionine [mg]	0.04	ns	0.01	ns
Phenylalanine [mg]	0.08	ns	0.07	ns
Cystine [mg]	0.09	ns	0.04	ns
Tyrosine [mg]	0.08	ns	0.03	ns
Threonine [mg]	0.06	ns	0.02	ns
Tryptophan [mg]	0.08	ns	0.02	ns
Valine [mg]	0.08	ns	0.01	ns
Arginine [mg]	0.06	ns	-0.01	ns
Histidine [mg]	0.05	ns	0.10	ns
Myristoleic acid [C14:1. g]	0.01	ns	0.06	ns
Pentadecanoic acid [C15:1. g]	0.14	ns	0.08	ns
Palmitoleic acid [C16:1. g]	-0.09	ns	0.05	ns
Heptadecenoic acid [C17:1. g]	0.11	ns	0.06	ns
Oleic acid [C18:1. g]	0.00	ns	0.04	ns
Eicosenoic acid [C20:1. g]	-0.15	ns	-0.03	ns
Erucic acid [C22:1. g]	-0.20	<0.05	0.00	ns
Total monounsaturated fatty acids [MUFA. g]	-0.01	ns	0.04	ns
Linoleic acid [C18:2. g]	0.03	ns	-0.04	ns
Alpha-linolenic acid (ALA) [C18:3. g]	-0.09	ns	-0.05	ns
Stearidonic acid [C18:4. g]	-0.23	<0.01	-0.07	ns
Arachidonic acid (AA) [C20:4. g]	-0.10	ns	0.02	ns
Eicosatrienoic acid [C20:3. g]	0.01	ns	0.05	ns
Eicosapentaenoic acid (EPA) [C20:5. g]	-0.21	<0.05	0.00	ns
Docosapentaenoic acid (DPA) [C22:5. g]	-0.22	<0.05	-0.01	ns
Docosahexaenoic acid [DHA. C22:6. g]	-0.24	<0.05	-0.04	ns
Total polyunsaturated fatty acids[PUFA. g]	-0.03	ns	-0.05	ns
Butyric acid [C4:0. g]	0.03	ns	0.03	ns
Caproic acid [C6:0. g]	0.03	ns	0.06	ns
Caprylic acid [C8:0. g]	-0.07	ns	0.03	ns
Capric acid [C10:0. g]	-0.07	ns	0.03	ns
Lauric acid [C12:0. g]	-0.05	ns	0.02	ns
Myristic acid [C14:0. g]	-0.04	ns	0.06	ns
Pentadecanoic acid [C15:0. g]	-0.01	ns	0.07	ns
Palmitic acid [C16:0. g]	-0.02	ns	0.07	ns
Heptadecanoic acid [C17:0. g]	0.00	ns	0.07	ns
Stearic acid [C18:0. g]	0.03	ns	0.07	ns
Arachidic acid [C20:0. g]	-0.04	ns	-0.02	ns
Saturated fatty acids: total [SFA. g]	-0.02	ns	0.07	ns
Sucrose [g]	0.13	ns	0.09	ns
Lactose [g]	0.12	ns	0.16	ns
Sodium [mg]	0.14	ns	-0.12	ns
Cholesterol [mg]	-0.09	ns	-0.04	ns
Energy [kcal]	0.13	ns	-0.09	ns

Water [g]	-0.06	ns	0.01	ns
Ash [g]	0.17	ns	-0.07	ns
Alanine [mg]	0.01	ns	0.02	ns
Aspartic acid [mg]	0.05	ns	-0.01	ns
Glutamic acid [mg]	0.09	ns	0.07	ns
Glycine [mg]	0.01	ns	0.02	ns
Proline [mg]	0.07	ns	0.08	ns
Serine [mg]	0.07	ns	0.01	ns
Starch [g]	0.15	ns	0.06	ns
Physical activity- Health related behaviours moderate (PA-HRBI)	0.23	<0.01	0.14	ns
Physical activity- health related behaviours hard(PA-HRBII)	0.19	<0.05	0.20	<0.05
Physical activity- Energy expenditure PA-EE [kcal/kg/day]	-0.01	ns	0.07	ns
